# Genomic organization of a Gamma-6 papillomavirus metagenomic discovered from vaginal swab samples of Chinese pregnant women

**DOI:** 10.1186/s12985-020-01319-9

**Published:** 2020-03-31

**Authors:** Yu Ling, Jianqiang Wang, Jun Yin, Jianpu Xu, Yufan Wu, Rui Zhou, Juan Lu, Shixing Yang, Xiaochun Wang, Quan Shen, Wen Zhang

**Affiliations:** 1 School of Medicine, Jiangsu University, 301 Xuefu Road, Zhenjiang, Jiangsu 212013 People’s Republic of China; 2 Department of Laboratory Medicine, Nanjing Maternity and Child Health Care Hospital, Nanjing, Jiangsu 210004 People’s Republic of China; 3 Intensive Care Unit, Jintan District Hospital of Traditional Chinese Medicine, 1006 Huixian Road, Changzhou, Jiangsu 213200 People’s Republic of China; 4 Zhangjiagang Customs, 28 Renmin Road, Zhangjiagang, Jiangsu 215699 People’s Republic of China

**Keywords:** Human papillomaviruses, Gamma-6 papillomavirus, Virus metagenomics, Complete genome, Genomic organization

## Abstract

A complete genome sequence of human papillomaviruses (HPV) named as HPV-ujs-21015 was determined by viral metagenomic and PCR methods. The complete genome is 7354 bp in length with GC content of 41.7%, of which the genome was predicted to contain six ORFs (Open Reading Frame, ORF) coding for four early proteins (E7, E1, E4, and E2) and two late proteins (L1 and L2). Phylogenetic analysis based on the complete genome and the L1 protein showed that HPV-ujs-21015 belongs to a type 214 member within genus Gamma-6 papillomavirus. It is the first complete genome of Gamma-6 papillomavirus discovered from pregnant women in China.

## Main text

Human papillomavirus (HPVs), a member of the *Papillomaviridae* family, are nonenveloped, double-strand circular DNA viruses with an approximately 8 kb genome in length. In the circular genome of HPV, eight genes are typically encoded. L1 and L2 code capsid proteins of virus, which can help virus entry into the basal layer keratinocytes [1, 2]. E2 protein is required for the transcription of viral genes and replication, and also recruits the viral DNA helicase E1 to keeping viral genomes in host cells [3]. E6 and E7 are believed to drive cellular immortalization and maintain the transformed phenotype during tumor progression, to exert functions by binding with many cellular protein to activate cancer hallmarks [4]. HPVs are classified into genera (alpha, beta, gamma, mu, and nu), species, types and even variants based on the nucleotide similarity, with the different types having different life-cycle characteristics and disease associations [5, 6]. HPV persistent infection is the main risk factor for the development of many tumors especially cervical tumor [7]. Although there were numerous ways to prevent the infection of HPV, such as vaccination, over 600,000 cases per year of cervical cancer were recorded worldwide [8]. According to the data from International HPV Reference Center at the Karolinska Institute, Stockholm, Sweden, as of May 6, 2016, two hundred and twenty-six reference HPV types, ranging from HPV-1 to HPV-226, were officially recognized (https://www.hpvcenter.se/human_reference_clones/). The determination of HPV genome can be helpful to understand the genomic characteristics and the clinical relevance of these new HPV strains. In recent years, in addition to frequently-used methods like PCR, some new methods including viral metagenomics were used to acquire the genome of HPV more efficiently [9, 10].

In our current study, the viral nucleic acid sequences from vaginal swabs were investigated through viral metagenomics. A total of 100 vaginal swabs were collected from the health pregnant women who visited hospital for antenatal follow-up of pregnancy in Shanghai City, China, in 2017. The total viral nucleic acid was isolated using QiaAmp Mini Viral RNA kit (Qiagen, USA) according to the manufacturer’s protocol after centrifugation, filtration and DNase and RNase digestion, as we described previously, and pooled into 10 libraries [9]. The produced nucleic acids (both DNA and RNA) were subjected to reverse transcript with N8 random primers (Sangon, Shanghai, China), and the second stand was generated using Klenow enzyme (NEB, Ipswich, USA). The libraries were then constructed by the Nextera XT DNA sample Preparation Kit (Illumina, CA, USA) following the protocol, and the prepared libraries were sequenced by Illumina Miseq platform with 250 bases paired ends with dual barcoding for each pool.

The total numbers of sequence reads generated for the 10 libraries were 73,264 (swab01), 45,462 (swab02), 100,518 (swab03), 111,398 (swab04), 82,612 (swab05), 436,560 (swab06), 903,618 (swab07), 71,544 (swab08), 273,046 (swab09), and 51,590 (swab10). Raw data were processed according to the standard procedure which included debarcoding, trimming and assembling [11]. Contigs and singlet reads were then matched against a customized viral proteome database using BLASTx with an E value cutoff of < 10^− 5^. Bioinformatics analysis was performed according to a previous study [9]. PCR and sanger sequencing were carried out to bridge the gaps between sequences as well as assess the prevalence of HPV strain identified in this study. Putative ORFs (Open Reading Frame, ORF) in the genome of HPV-ujs-21015 were predicted by Geneious Prime software (version 2020.0.4). The closest viral strains based on best BLASTx hits and the representative members of species and genera were selected to perform the phylogenetic analyses (Table 1). In order to construct the phylogenetic tree, sequence alignment was performed using Clustal W with the default settings. Phylogenetic tree was generated using the maximum likelihood method based on Jones-Taylor-Thornton (JTT) model by MEGA 7.0 with 1000 bootstrap. Bootstrap values for each node are given in the trees.

**Table 1:** The reference HPV strains and their genera and species. Classification was based on International Committee on Taxonomy of Viruses (https://talk.ictvonline.org/ictv-reports/ictv_online_report/dsdna-viruses/w/papillomaviridae), International HPV Reference Center at the Karolinska Institute, Stockholm, Sweden (http://www.hpvcenter.se), and Bernard et al., *Virology*. 2010 May 25; 401(1): 70–79

genus	species	abbreviation	GenBank accession number
alphapapillomavirus	alpha-1	HPV32	NC_001586
	alpha-2	HPV3	X74462
	alpha-3	HPV61	NC_001694
	alpha-4	HPV2	NC_001352
	alpha-5	HPV26	NC_001583
	alpha-6	HPV30	NC_038889
	alpha-7	HPV18	NC_001357
	alpha-7	HPV85	AF131950
	alpha-8	HPV7	NC_001595
	alpha-9	HPV16	NC_001526
	alpha-9	HPV58	D90400
	alpha-10	HPV6	NC_001355
	alpha-11	HPV34	NC_001587
	alpha-13	HPV54	NC_001676
	alpha-14	HPV71	NC_039089
betapapillomavirus	beta-1	HPV5	NC_001531
	beta-2	HPV9	NC_001596
	beta-2	HPV23	U31781
	beta-3	HPV49	NC_001591
	beta-4	HPV92	NC_004500
	beta-5	HPV96	NC_005134
	unclassified	isol Ki88	EU410347ACC78262
gammapapillomavirus	gamma-1	HPV4	NC_001457
	gamma-1	HPV95	AJ620210
	gamma-2	HPV48	NC_001690
	gamma-3	HPV50	NC_001691
	gamma-4	HPV60	NC_001693
	gamma-5	HPV88	NC_010329
	gamma-6	HPV101	NC_008189
	gamma-6	mw03c65	MF588697
	gamma-7	HPV109	NC_012485
	gamma-8	HPV112	NC_012486
	gamma-9	HPV116	NC_013035
	gamma-10	HPV121	NC_014185
	gamma-11	HPV126	NC_016157
	gamma-12	HPV127	NC_014469
	gamma-12	HPV132	GU117632
	gamma-12	HPV148	GU129016
	gamma-12	HPV199	KJ913662
	gamma-13	HPV128	NC_014952
	gamma-14	HPV131	NC_014954
	gamma-15	HPV135	NC_017993
	gamma-16	HPV137	NC_017995
	gamma-17	HPV144	NC_017997
	gamma-18	HPV156	NC_033781
	gamma-19	HPV161	NC_038522
	gamma-19	HPV162	JX413108
	gamma-19	HPV166	JX413104
	gamma-20	HPV163	NC_028125
	gamma-21	HPV167	NC_022892
	gamma-22	HPV172	NC_038523
	gamma-23	HPV175	NC_038524
	gamma-24	HPV178	NC_023891
	gamma-24	HPV197	KM085343
	gamma-25	HPV184	NC_038914
	gamma-27	HPV201	NC_027528
	unclassified	HPV-ZJ01	KX082661
	unclassified	isol CH2	KF791917
	unclassified	isol Fi864	KC311731
	unclassified	isol KC5	JX444073
mupapillomavirus	mu-1	HPV1	NC_001356
	mu-2	HPV63	NC_001458
	mu-3	HPV204	NC_038525
nupapillomavirus	nu-1	HPV41	NC_001354

## Results and discussion

**Fig. 1:**
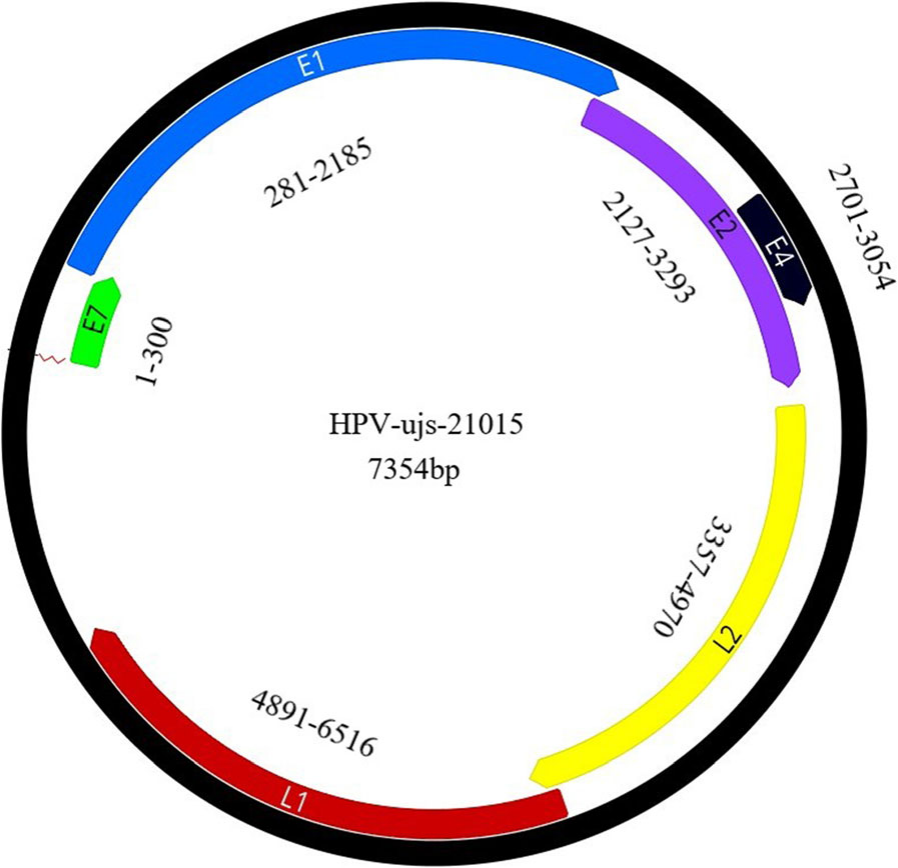
Genomic organization of HPV-ujs-21015. The genomic positions of viral genes (E7, E1, E2, E4, L1 and L2) were indicated in the figure

**Fig. 2:**
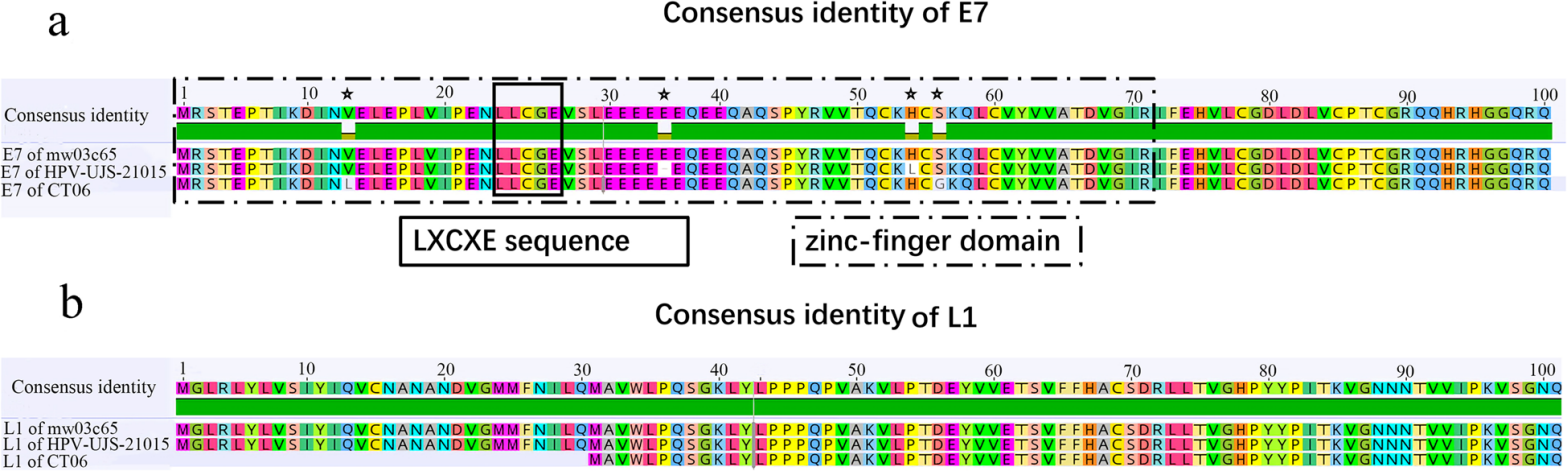
Alignment of amino acid of E7 (**a**) and L1 proteins (**b**) between HPV-UJS-2015 and Related HPVs. The LXCXE and Zinc-finger domains were enclosed with solid or dotted box, respectively. Mutation and deletion were marked with solid or blank stars

A strain of HPV named as HPV-ujs-21015 (GenBank accession no. MN400665, see Additional file 1) was determined in the vaginal swab (containing 1654 reads in library swab02), of which the complete genome is 7354 bp in length with GC content of 41.7%. The genome of HPV-ujs-21015 was predicted to contain six ORFs coding for four early proteins (E7, E1, E4, and E2) and two late proteins (L1 and L2) (Fig. 1). The nucleic acid lengths of these proteins were 300, 1905, 354, 1167, 1626 and 1614, respectively, and the positions on the genome were showed in Fig. 1. Notably, the E6 gene that plays a crucial role in the cell transformation through binding of p53 tumor suppressor protein was absent in this strain, which was consistence with other HPV214 strains [12, 13]. E6 as well as E7 is believed to be directly responsible for the development of HPV-induced carcinogenesis. In the high risk HPVs, they do this cooperatively by targeting diverse cellular pathways including the regulation of cell cycle control. Meanwhile, there is a view that the lost function of E6 in HPV214 may be compensated for in its E7 protein which has an LXCXE (Fig. 2a) motif that has been shown to bind pRB in HPV16 and other high risk HPV types.

According to the International Committee on Taxonomy of Viruses (ICTV), a viral type within a species has 71 to 89% identity with other types within the same species based on the comparative homology of the L1 DNA sequence. Additionally, there are several subtypes and variants within a type, which share 90 to 98% and more than 98% identity, respectively. In the current study, sequence analysis indicated that HPV-ujs-21015 shared the highest nucleotide (nt) sequence identity (99%) with a type 214 strain named CT06 isolated from South African strain (GenBank no. MF509819), as well as strain mw03c65 (GenBank accession no. MF588697), which was an unclassified strain detected in patients with immunodeficiency in USA.

Similar to mw03c65 and CT06 strain, the putative E7 protein of HPV-ujs-21015 strain contained one zinc-finger domain and an LXCXE sequence (Fig. 2a), which is critical for transforming activities by way of binding a number of important cellular regulatory proteins, including tumor suppressor: Retinoblastoma protein (pRb). Compared with these two strains, HPV-ujs-21015 had one amino acid deletion and three mutations (Fig. 2a). Whether the deletion and mutations affect the biological function of E7 will require more research. Intriguingly, another protein with significant diversity was L1, of which HPV-ujs-21015 had the 100% amino acid similarity with mw03c65, but was thirty consensus amino acid longer than that of CT06 strain in the 5’end (Fig. 2b).

**Fig. 3:**
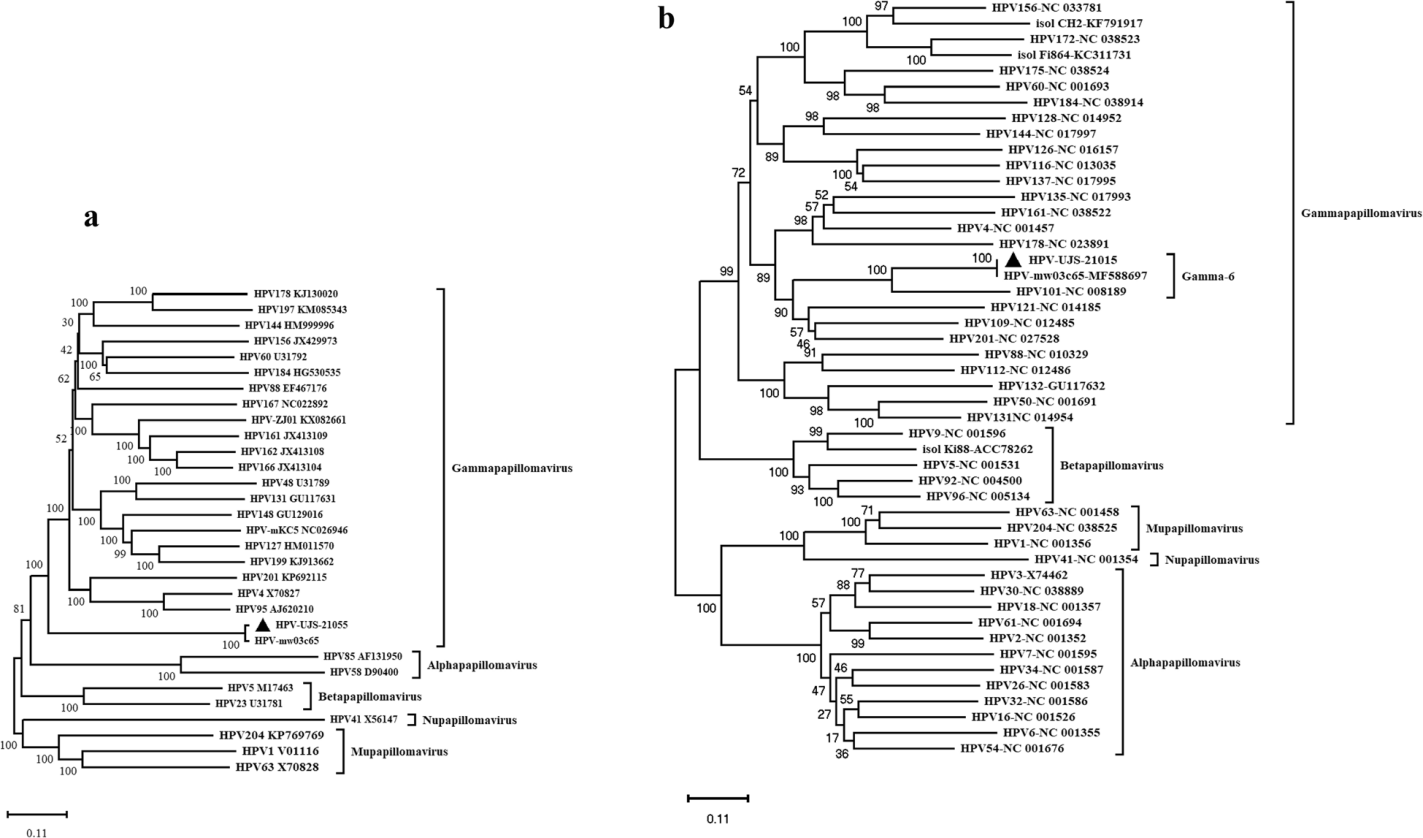
Phylogenetic trees constructed based on the complete genome (**a**) and L1 protein (**b**) were constructed, respectively, using maximum-likelihood method by MEGA-X with 1000 bootstrap. GenBank accession nos. of the reference strains and their abbrevation were showed in the trees. The strain determined in this study was marked with a triangle

To characterize the phylogenetic relationship between HPV-ujs-21015 and related HPV reference strains, two phylogenetic trees based on the complete genome and L1 protein were constructed, respectively, by MEGA 7.0. Both trees revealed that the reference HPVs were clustered well in their genera and types. The phylogenetic tree based on the complete genome showed that HPV-ujs-21015 belonged to Gamma-papillomavirus (Fig. 3a). The other phylogenetic tree based on the L1 protein further assigned HPV-ujs-21015 within the group of type 214 in Gamma-6, being closely related to mw03c65 strain (Fig. 3b). In summary, our results suggest that all of these three strains isolated from different countries were variants with the genotype 214.

HPVs comprise five evolutionary groups with different epithelial tropisms and disease associations. Traditionally, based on the location of the certain virus genome was found, HPVs have also been classified as mucosal or cutaneous types [1]. Increasing evidences revealed that Gamma-PVs showed broad tissue tropism, with the detection locations ranging from health skin and cutaneous lesions to genital lesions [10, 14, 15]. DNA of some Gamma-PVs types were detected in skin cancer raised concerns of some Gamma-PVs associations with cancers, especially in patients with immunodeficiency or immunosuppression [16, 17]. In the current study, HPV-ujs-21015 strain was identified from a health pregnant woman who visited hospital for antenatal follow-up. Vaginitis or other vaginal disease were not found by the attending gynecologist. Generally, both mucosal or cutaneous disease relied on the persistent infection of HPVs. Therefore, whether the infection of HPV-ujs-21015 can cause disease or not is still unknown. A total of one hundred of vaginal swab samples from health pregnant women who visited hospital for antenatal follow-up were screened by PCR method with a set of nested primers (data not showed) designed on HPV-ujs-21015 L1 gene. Result showed that two samples were positive (2/100). The prevalence and disease association of HPV-ujs-21015 need to be clarified through larger sample size, biological and histological experiments.

In conclusion, we determined and characterized the complete genome sequence of a genotype 214 Gamma-6 papillomavirus, which was isolated from a health pregnant woman of China. To the best of our knowledge, it is the first complete genome of Gamma-6 papillomavirus detected in Pregnant Women of China.

## Supplementary information

**Additional file 1.** The complete genome of HPV-ujs-21015.

## Data Availability

The sequences of full-length envelope gene generated in this study have been deposited in GenBank under the accession numbers MN400665.

## Abbreviations

aa: Amino acid
HPV: Human papillomaviruses
ORF: Open Reading Frame
ICTV: International Committee on Taxonomy of Viruses
nt: Nucleotide
pRb: Retinoblastoma protein
