# Supplementary material for: Genomic organization of a Gamma-6 papillomavirus metagenomic discovered from vaginal swab samples of Chinese pregnant women
Source: Virol J. 2020 Mar 31;17:44. doi: 10.1186/s12985-020-01319-9 (PMC7110641; doi:10.1186/s12985-020-01319-9)
Supplement: Supplementary file 1 — Additional file 1. The complete genome of HPV-ujs-21015. [file 12985_2020_1319_MOESM1_ESM.zip › Additional file 1 legend.docx]

Supplementary material 1. The complete genome of HPV-ujs-21015.
